# Effectively Enhancing the Physiological Activity and Sensory Quality of Whole Calamondin Puree via Yeast Fermentation

**DOI:** 10.3390/ijms252211984

**Published:** 2024-11-07

**Authors:** Hongjian Zhang, Shuaiguang Liu, Zewei Ma, Huan Huang, Lianhe Zheng, Yan Tian, Qiuping Zhong

**Affiliations:** 1 Hainan University-HSF/LWL Collaborative Innovation Laboratory, College of Food Sciences & Engineering, Hainan University, Haikou 570228, China; 2 Hainan Institute of Grain and Oil Science, Qionghai 571400, China; 3 Engineering Research Center of Utilization of Tropical Polysaccharide Resources (Ministry of Education), College of Food Science and Engineering, Hainan University, Haikou 570228, China

**Keywords:** *Citrus microcarpa*, yeast fermentation, phenolic compounds, flavor compounds, metabolic pathways

## Abstract

To explore the feasibility of pure yeast fermentation in whole Calamondin puree (FWCP) for the utilization of the whole fruit, yeasts were isolated from naturally fermented Calamondin, and their fermentation characteristics were evaluated. The results indicated that all yeasts were able to ferment FWCP, reducing the sour taste by degrading citric acid, increasing the contents of nutrients (such as phenols and limonins) and volatile compounds, and enhancing the antioxidant activity and inhibition of α-glucosidase activity (*p* < 0.05). Among them, *P. terricola* QJJY1 and *H. opuntiae* QJJY14 exhibited stronger abilities to degrade organic acids, with *P. terricola* QJJY1 enhancing the antioxidant capacity by releasing phenolic compounds such as phloretin-3′,5′-di-c-β-glucoside, vitexin-2″-O-rhamnoside, and isomargaritene. Moreover, *H. opuntiae* QJJY15 improved the contents of characteristic volatile compounds such as terpene hydrocarbons and higher alcohols. In total, 70 components were identified as differential metabolites based on their fold change in the metabolites, with 42 differential metabolites involved in 29 metabolic pathways across four strains. The main pathways related to phenol and flavor enrichment were flavonoid, flavone, and flavonol biosynthesis, monoterpenoid biosynthesis, and glyoxylate and dicarboxylate metabolism. Therefore, yeast fermentation is an effective method for utilizing whole Calamondin.

## 1. Introduction

Calamondin (*Citrus macrocarpa*) is a hybrid of *Citrus reticulate* Blanco and *Fortunella* spp. [1]. Calamondin has attracted more attention due to its unique flavor and rich contents of nutrients such as phenols, limonins, and vitamin C [2]. At present, juicing is the main way to utilize whole Calamondin. However, this method results in very low utilization of the whole fruit, producing a large number of by-products (approximately 65% of the fruit’s total weight). Studies have shown that Calamondin is rich in pectin, carbohydrates, cellulose, crude protein, and trace elements [2], which can be used as natural fermentation media for microorganisms. Moreover, Calamondin contains abundant phenolic compounds, such as phloretin-3′,5′-di-c-β-glucoside (GDPP) and hesperidin [3], which have good physiological functions such as antioxidant activity, lowering blood sugar and lipids, and lowering cholesterol [4]. However, phenolic compounds are often covalently bonded to components in the plant cell wall (such as hemicellulose, pectin, and structural proteins) or linked to polysaccharides, resulting in low bioavailability [5]. Therefore, developing a new processing method to improve whole-fruit utilization and enrich its nutritional value and flavor is necessary.

Fermentation can improve the sensory quality and prolong the shelf life of products. Microorganisms can promote the conversion of insoluble bound phenols into soluble phenols in citrus by releasing hydrolases, thus improving biological activity [6,7,8]. The utilization of starter cultures can reduce fermentation time, improve product characteristics, and maintain product quality stability [9]. Strains derived from the fruits themselves tend to have stronger viability and better fermentation characteristics, so can enhance the quality of fermented products [9]. Thus, the microorganisms naturally present in Calamondin may serve as potential strain resources for bioprocessing technologies.

Among the microorganisms screened, the range of bacterial fermentation substrates is relatively narrow, limited mainly to glucose and fructose. In contrast, yeast can not only utilize low-molecular-weight sugars but also metabolize polysaccharides such as cellulose, starch, and pectin [10,11]. Additionally, bacterial fermentation is more sensitive to environmental conditions (e.g., temperature and pH), increasing the difficulty of production, while yeasts are more relaxed in the production environment, making it easier to achieve stable quality control in industrial production [12]. Yeast fermentation also produces a greater variety of fermentation products, improving both flavor and nutritional value more effectively than bacterial fermentation [9,11]. According to reports, yeasts have attracted more attention compared to lactic acid bacteria (LAB) due to their better survival ability, safety, and fermentation characteristics [10]. Yeasts show strong acid resistance and can metabolize organic acids such as citric acid, thereby reducing the sour taste of produce [13]. They can also metabolize and release active substances such as carotenoids, tocopherols, ascorbic acid, and phenols, which play a vital role in antioxidant activity [14]. In addition, yeasts can be used as a biotechnological tool to increase the bioaccessibility of phenolics and improve the volatile profile of fruits [9].

Currently, most studies have focused on the fermentation of citrus juice [15], residue [7], and peel [16] using LAB or other bacteria; however, research on whole citrus fruit fermentation is still limited. Moreover, the pH value of whole Calamondin puree is only about 3.0, and the organic acid content is significantly higher than that of other citrus, such as mandarin [8] and Changshan-huyan [16]. Therefore, the microorganisms contained in the natural fermentation process of whole Calamondin puree may have better metabolic activity in adapting to extreme environments such as low pH, metabolizing organic acids, and releasing nutrients and flavor substances. At present, there is no report on the selection and evaluation of fermentation characteristics of strains from Calamondin for whole-fruit fermentation.

Therefore, the purposes of this study were (1) to isolate and identify yeasts from naturally fermented Calamondin; (2) to compare the fermentation characteristics of these yeasts, including the basic physicochemical properties, nutrient content, volatile compounds, functional activity, and sensory quality of FWCP; and (3) to predict the metabolic pathways during yeast fermentation. Thus, this study could help with exploring the feasibility of using yeast as a biotechnology tool to optimize the utilization of whole citrus fruits.

## 2. Results and Discussion

### 2.1. Yeast Isolation and Identification

**Figure 1:**
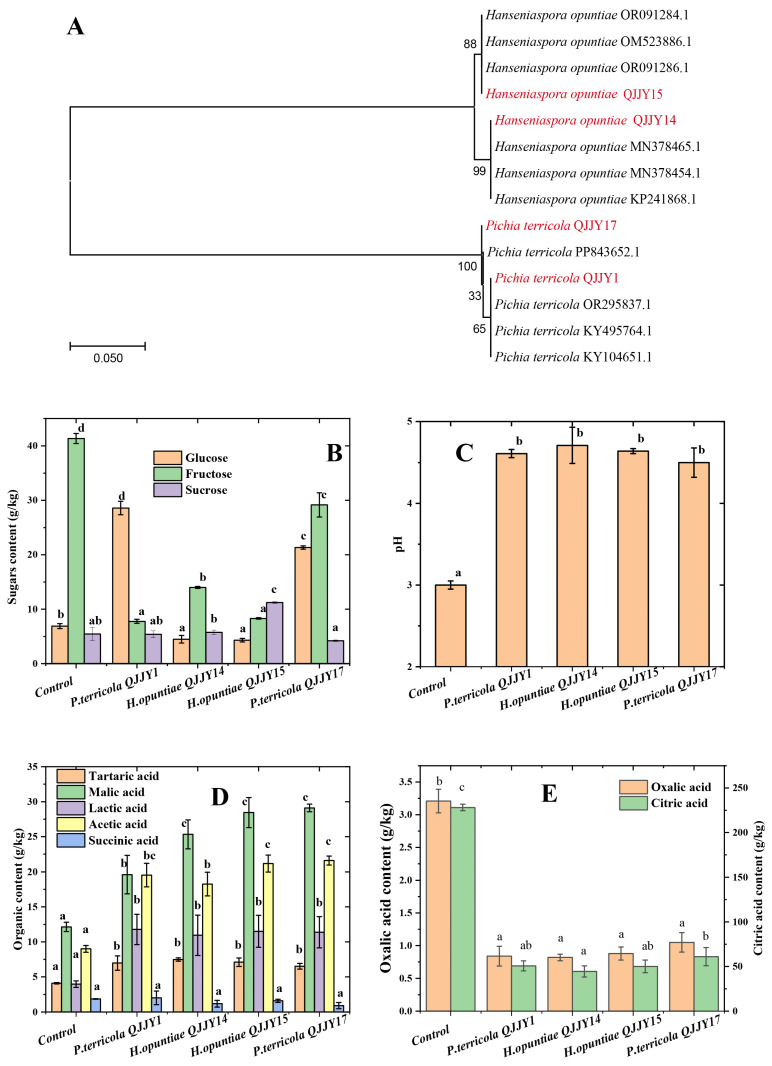
Phylogenetic tree of four yeast strains (**A**), sugar content (**B**), pH values (**C**), organic acid content (**D**), and oxalic and citric acid content (**E**) in nonfermented (control) and fermented whole Calamondin puree (20 days) using *P. terricola* QJJY1, *H. opuntiae* QJJY14, *H. opuntiae* QJJY15, and *P. terricola* QJJY17. Different lowercase letters in the figure indicated that there were significant differences in physicochemical indexes of different fermented samples (*p* < 0.05).

Twenty-four strains of yeast were screened from naturally fermented Calamondin using YPD medium. The pure yeast strains were inoculated into the sterilized whole Calamondin puree to rescreen for viability. The count of four yeast strains was more than 10^8^ CFU/mL, indicating good viability. The colonies of the four yeasts were milky white, round, and slightly convex, with a viscous texture, making them easy to pick on solid YPD medium. The cell morphology was elliptical upon microscopic observation. The phylogenetic tree constructed based on ITS sequencing is shown in Figure 1A. The four strains were all non-Saccharomyces yeast, of which two strains were *Pichia terricola* and two strains were *Hanseniaspora opuntiae*. In particular, *P. terricola* QJJY1 and *P. terricola* OR 295837.1 were the closest, with 65% similarity, and *P. terricola* QJJY17 and *P. terricola* PP 843652.1 were the closest, with 100% similarity. *H. opuntiae* QJJY14 and *H. opuntiae* MN 378465.1 were the closest, with 99% similarity, and *H. opuntiae* QJJY15 and *H. opuntiae* OR091286.1 were the closest, with 88% similarity.

*H. opuntiae* and *P. terricola* are considered safe because they do not produce gelatinase, hemolytic, or DNAse activity. Moreover, *H. opuntiae* and *P. terricola* improve the flavor and nutritional content of food [10]. Thus, *H. opuntiae* and *P. terricola* isolated from naturally fermented Caatinga fruits could significantly increase the phenolic bioavailability and improve the flavor characteristics of fermented soursop and umbu-caja’ pulp [9]. In conclusion, *H. opuntiae* and *P. terricola* exhibited the potential for enhancing the flavor and nutrition of food. Therefore, the above strains were used for fermentation of whole Calamondin puree to evaluate their ability to improve the quality of FWCP.

### 2.2. pH, Sugar, and Organic Acid Profile of the Fermented Fruit Puree

The sugar, organic acid, and pH values in the whole-fruit puree before and after fermentation (20 days) of the four yeast strains are shown in Figure 1B–E. The fructose content significantly decreased (*p* < 0.05) while the sucrose content showed no significant changes (except for samples with *H. opuntiae* QJJY15) after fermentation. Moreover, the glucose content significantly increased after fermentation with *P. terricola* QJJY1 and *P. terricola* QJJY17 but significantly decreased after fermentation with *H. opuntiae* QJJY14 and *H. opuntiae* QJJY15 (*p* < 0.05). *P. terricola* can synthesize glucose by degrading cellulose through the secretion of cellulose and β-glucosidase [9], which may be the main reason for the increased glucose content in FWCP.

**Figure 2:**
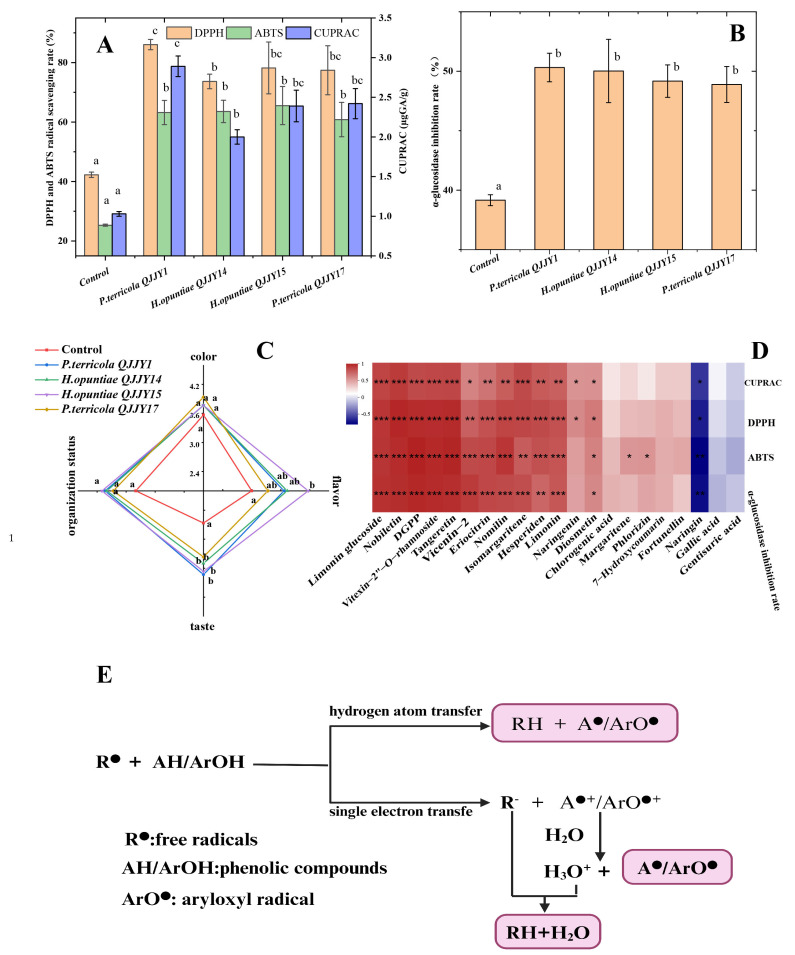
Antioxidant activity (**A**), α-glucosidase inhibition rate (**B**), and sensory analysis (**C**) of nonfermented (control) and fermented whole Calamondin puree (20 days) using *P. terricola* QJJY1, *H. opuntiae* QJJY14, *H. opuntiae* QJJY15, and *P. terricola* QJJY17. Correlation heatmap of the nutritional components and functional activity (**D**). Antioxidant mechanism of phenolic compounds (**E**). Different lowercase letters in the figure indicated that there were significant differences in physicochemical indexes of different fermented samples (*p* < 0.05). “*” indicates *p* < 0.05, “**” indicates *p* < 0.01, “***” indicates *p* < 0.001.

The pH value of FWCP significantly increased from 3.00 to 4.50 after fermentation (Figure 1C). The citric acid (228.11 g/kg) content was the highest of the organic acids in Calamondin, and its content decreased in the range of 73.28–80.51% after fermentation with the four strains of yeast (Figure 1E). The high citric acid content in fruit is the main cause of its sour taste and poor edibility [13]. Yeasts possess carboxylic acid transporters for citric acid uptake [13], which can metabolize citric acid as an important energy source via the tricarboxylic acid cycle (TCA), reducing the citric acid content. Thus, yeast fermentation can effectively reduce the citric acid content, improving the taste of FWCP (Figure 2C). *H. opuntiae* QJJY14 showed a stronger ability to reduce the acid content than *P. terricola* QJJY17 (*p* < 0.05), but there was no significant difference between *P. terricola* QJJY1 and *H. opuntiae* QJJY15.

The lactic, acetic, malic, and tartaric acid contents increased in the ranges of 175.57–196.47%, 102.89–139.89%, 61.45–139.79%, and 82.68–59.02%, respectively, after fermentation. However, the succinic acid content did not significantly change. Studies have shown that the lactic, acetic, and tartaric acid contents in fermented products are positively correlated with DPPH and ABTS free radical scavenging [17]. Therefore, an increase in organic acid content may promote the antioxidant ability of FWCP because yeast can synthesize lactic and acetic acid by consuming sugars such as fructose and sucrose [18]. In this study, *P. terricola* more effectively synthesized lactic and acetic acid and increased their contents than *H. opuntiae*, which may have been related to the greater ability of *P. terricola* to metabolize sugars.

The principal component analysis (PCA) explained 83.8% of the variability in the organic acids and sugars (PC1: 68.9%, PC2: 14.9%) (Supplement Figure S1A). Fermented fruit puree, located on the right in Figure S1A, was characterized by higher concentrations of tartaric acid, malic acid, lactic acid, acetic acid, and glucose. The control sample, located on the left in Figure S1A, was characterized by higher concentrations of citric acid, oxalic acid, and fructose. In conclusion, yeast fermentation can change the sugar and organic acid contents in FWCP, which may reduce the sour taste caused by a high citric acid content.

### 2.3. Free Amino Acid Profile of the Fermented Fruit Puree

The contents of 18 kinds of free amino acids in whole Calamondin puree before and after fermentation are shown in Table 1. Although the total free amino acid content significantly decreased (*p* < 0.05), fermentation using the four yeasts led to increased leucine (Leu) and tryptophan (Trp) contents in the range of 33.34–111.11% and 200–260%, respectively, in all samples. The decrease in free amino acid content may have been related to its metabolism toward secondary metabolites, such as alcohols, acids, and esters, as reflected by the significant increases in the alcohol and ester contents in FWCP (Table 2 and Supplementary Figure S2) [19]. Moreover, yeast fermentation can increase the amino acid content in fermented products by releasing protease to degrade proteins [20], which may be the reason for the increased Leu and Trp contents in FWCP. Leucine (Leu) and tryptophan (Trp), as essential amino acids for humans, can prevent muscle loss by quickly metabolizing to glucose [21] and act as precursors of 5-hydroxytryptamine, which plays a vital role in regulating mental rhythms and improving sleep in the human body [22]. In this study, *H. opuntiae* QJJY14 showed a better ability to synthesize Leu and Trp compared to the other three strains of yeast. Therefore, samples fermented with *H. opuntiae* QJJY14 may better improve sleep and prevent damage to muscle tissue.

The principal component analysis (PCA) explained 89.1% of the variability in the free amino acids (PC1: 70.4%, PC2: 18.7%) (Supplement Figure S1B). Fermented fruit puree, located on the left of the figure, was characterized by higher concentrations of Trp, Leu, and Cys. The control sample, located on the right of the figure, was characterized by higher concentrations of Ser, Pro, Gly, Phe, Tyr, and Asp. In conclusion, yeast fermentation can significantly change the free amino acid profile of FWCP, and the strain used has a specific effect on its composition.

### 2.4. Phenolic Compound and Limonoid Profile of Fermented Fruit Puree

The contents of 18 kinds of phenolic compounds and 3 kinds of limonoids in whole Calamondin puree are shown in Table 3. Compared to those in the control, the total phenol and limonin contents were significantly increased in the samples fermented with the four kinds of yeast, which increased in the ranges of 47–52% and 21.1–30.1%, respectively. However, there was no significant difference between the total polyphenol and limonin contents in the four samples. The increased phenolic content implied an increased antioxidant capacity of the samples after fermentation.

In particular, nine kinds of phenolic compounds (vicenin-2, vitexin-2″-O-rhamnoside, eriocitrin, DGPP, eriocitrin, hesperidin, isomargaritene, nobiletin, and tangeretin) and three kinds of limonins (limonin glucoside, limonin, and nomilin) significantly increased (*p* < 0.05) after fermentation. Moreover, *P. terricola* QJJY1 exhibited a stronger ability to release the main phenolic, such as DGPP, vitexin-2″-O-rhamnoside, isomargaritene, or nobiletin, than the other three strains of yeast. Among them, the DGPP and limonin contents, as the main phenolic compound and limonoid in Calamondin, were higher than 50% and 33%, respectively. Investigations have indicated that DGPP and limonin can improve obesity by inhibiting the accumulation of intracellular fat and triglycerides [23] and reducing cholesterol by regulating the ApoB content [24]. These results show that yeast-fermented whole Calamondin puree can play a role in reducing body weight and regulating cholesterol.

A better visualization of the data is provided in the PCA map in Supplementary Figure S1C. The PCA explained 82.5% of the variability in the nutrients (PC1: 67.8%, PC2: 14.7%). Fermented and unfermented samples can be clearly distinguished. The unfermented samples are on the left, with a high naringenin content. The fermented samples, located on the right, have high DGPP, tangeretin, hesperidin, nomilin, vicenin-2, and vitexin-2″-O-rhamnoside contents. In addition, the strains had a specific effect on the composition of the nutrients. The samples fermented using *P. terricola* QJJY1 were in the first quadrant, and the naringin, diosmetin, and isomargaritene contents were higher. *P. terricola* QJJY17 and *H. opuntiae* QJJY15 were in the fourth quadrant, and chlorogenic acid, margaritene, and fortunellin contents were higher. In conclusion, yeast fermentation can promote the release of phenols and limonins and has great potential for improving the antioxidant capacity of FWCP and preventing obesity.

### 2.5. Volatile Compound Profile of the Fermented Fruit Puree

The concentrations of the volatile compounds in the whole Calamondin puree before and after fermentation are shown in Table 2. Yeast fermentation led to a total of 39 kinds of volatile compounds being identified by means of GC/MS, including 19 kinds of terpene hydrocarbons, 10 kinds of higher alcohols, 5 kinds of esters, 3 kinds of aldehydes, and 1 ketone. Furthermore, *P. terricola* QJJY1, *H. opuntiae* QJJY14, *H. opuntiae* QJJY15, and *P. terricola* QJJY17 produced 36, 34, 34, and 34 volatile compounds, respectively. Moreover, fermentation with the four strains of yeast increased the total volatile compound content in all samples (Supplementary Figure S2A). In particular, the total volatile compound content in the samples fermented using *H. opuntiae* QJJY15 was the highest. The above results show that yeast fermentation can change the volatile compound species in products and enhance the flavor of whole Calamondin puree.

In particular, the D-limonene, β-myrcene, α-pinene, and β-pinene contents, which are regarded as the most abundant terpene hydrocarbons and volatile compounds in Calamondin, significantly increased (Supplementary Figure S2E), indicating that fermentation endowed the whole Calamondin puree with the more characteristic flavor of “citrus, cedarwood, pine, and balsamic” [25]. The increased terpene hydrocarbon content may have increased for two reasons. First, yeast can secrete key enzymes involved in the metabolism of the mevalonate pathway, such as isoprene synthase, to synthesize terpene hydrocarbons [26]. Second, some nonsaccharomyces cerevisiae can hydrolyze glycosides to release terpene hydrocarbons by β-glucosidase and exo-1,3-glucanase [27]. Moreover, the terpene hydrocarbon content in the samples fermented using *H. opuntiae* was higher than in those of *P. terricola* and *H. opuntiae* QJJY15, indicating differences between the various bacterial species [27].

Compared to that in the control, the higher alcohol content significantly increased by approximately 84.34–109.89% (*p* < 0.05) (Supplementary Figure S2B), and there were no significant differences among the four yeast strains (*p* < 0.05).

2-Methyl-3-buten-2-ol, linalool, β-terpineol, terpinen-4-ol, and α-terpineol are regarded as the main higher alcohols in Calamondin puree, and their significantly increased contents after fermentation suggest that fermentation by yeast could enhance the “herb, coriander, wood, anise” odor of the whole puree. Moreover, newly formed alcohols, such as ageratriol and cis-geraniol, endowed the puree with a “sweet” odor. The increased higher alcohol content after fermentation may have occurred for two reasons: (1) yeast fermentation could degrade amino acids through the Ehrlich pathway [9], as reflected by the reduction in free amino acid content during fermentation (Table 1); (2) yeast fermentation could metabolize sugars through the de novo biosynthesis of amino acids [28].

After fermentation, the aldehyde content reduced by almost 90% with *P. terricola* QJJY1 and the aldehydes disappeared altogether after fermentation with the other yeasts. The reason for the decrease may have been that the aldehydes were converted into the corresponding alcohols or acids under the action of aldehyde dehydrogenase and alcohol dehydrogenase, which yeasts release [29].

Compared to those in the control, the ester contents in the samples fermented with *P. terricola* QJJY1 were significantly increased (*p* < 0.05), but there were no significant differences among the other three samples (Supplementary Figure S2D). The geranyl isovalerate and geranyl propionate contents, which are regarded as the main ester compounds in FWCP, significantly increased, which endowed the Calamondin puree with a “banana, floral, fruit” odor. Additionally, their contents in the samples fermented with *P. terricola* QJJY1 significantly increased, but those in the samples fermented with the other three yeasts significantly decreased (*p* < 0.05). Furthermore, fermentation with *H. opuntiae* QJJY14, *H. opuntiae* QJJY15, and *P. terricola* QJJY17 produced isoamyl acetate, which endowed the Calamondin puree with the aroma of banana. The change of esters may be related to esterification and alcoholysis. The esterification synthesis pathway is responsible for the formation of esters from acids and alcohols via dehydration under the catalysis of esterase and lipase. Alcoholysis is a synthetic pathway in which acyl groups of esters are transferred to alcohols under the catalysis of acyltransferase to form new esters and alcohols [30]. In general, the ester synthesis of *P. terricola* QJJY1 was superior to that of the other yeasts.

A better visualization of the data is provided in the PCA map in Supplementary Figure S1D. The PCA explained 87.7% of the variability in the volatile compounds (PC1: 57.4%, PC2: 30.3%). Fermented and control samples could be clearly distinguished. The control samples, located in the third quadrant, were characterized by higher concentrations of nonanal, decanal, and (E)-2-decenal, with a “fat, orange peel and tallow” odor. The fermented samples are located on the right. The samples with *P. terricola* QJJY1, located in the first quadrant, were characterized by higher concentrations of 1-nonanol, trans-farnesol, and geranyl propionate, with a “floral, mugue” odor. The samples with *H. opuntiae* QJJY14, *H. opuntiae* QJJY15, and *P. terricola* QJJY17, located in the fourth quadrant, were characterized by higher concentrations of isoamyl acetate, β-phellandrene, β-myrcene, and β-acorenol, with a “banana, mint, fruit” odor. In conclusion, fermentation can promote the release of volatile compounds and improve the aroma of whole Calamondin puree.

### 2.6. Sensory Analysis

Figure 2C shows the sensory evaluation scores for the whole Calamondin puree before and after fermentation. The sensory scores for the fermented samples for taste, flavor, color, and organization status were higher than those of the control, and the total scores for the samples fermented using *P. terricola* QJJY1(15.13), *H. opuntiae* QJJY14 (15.00), and *H. opuntiae* QJJY15 (15.7) were significantly higher than those for the control (12.63) (*p* < 0.05). After fermentation, the taste scores of all fermented samples significantly increased compared to that of the control due to the reduced sour taste, attributed to the citric acids metabolized by the yeasts (*p* < 0.05) [13]. The fermented samples had a stronger characteristic odor and showed a special fermentation aroma. The samples fermented using *H. opuntiae* QJJY14 had the highest flavor score, which was significantly better than that of the control (*p* < 0.05). The improvement in flavor may have been related to the new synthetic compounds (isoamyl acetate and cis-geranio) and the increase in total volatile content (Table 3). However, there was no significant difference in color or organization status between the fermented samples and the control (*p* > 0.05). In general, yeast fermentation can effectively reduce the sour taste, enhance the flavor, and improve the sensory quality of whole Calamondin puree.

### 2.7. Evaluation of Antioxidant Activity and α-Glucosidase Inhibition Rate

Scavenging free radicals in the body can effectively prevent oxidative-stress-related diseases such as aging, cardiovascular disease, and atherosclerosis [31]. Furthermore, the inhibition of α-glycosidase activity plays a vital role in reducing oligosaccharide/disaccharide digestion, delaying the absorption of blood sugar and reducing the risk of diabetes mellitus type 2 (T2DM) [32]. The antioxidant activity and α-glucosidase inhibition rate of the whole Calamondin puree before and after fermentation are shown in Figure 2A,B. Compared to those in the control, the antioxidant activity (DPPH, ABTS, and CUPRAC) and α-glucosidase inhibition rate of the fermented whole puree significantly increased (*p* < 0.05). In particular, *P. terricola* QJJY1 had an advantage over the other three strains of yeast in improving antioxidant activity and inhibiting enzyme activity. Similar results were also reported in the solid-state fermentation of pomelo peel using *Aspergillus oryzae* [33]. A correlation heatmap of nutrients and functional activities was drawn (Figure 2D), and their color-coded scale was graded from red to blue, representing a shift in correlation from positive to negative. The results indicate that the increased contents of compounds such as limonin glucoside, nobiletin, DGPP, vitexin-2″-O-rhamnoside, vicenin-2,eriocitrin, nomilin, tangeretin, isomargaritene, hesperidin, limonin, and diosmetin positively correlated with the antioxidant capacity and α-glucosidase inhibition rates.

Studies have shown that phenolic compounds such as vicenin-2, nobiletin, and hesperidin can bind to enzymes via noncovalent bonds and induce changes in their conformational structure, thereby inhibiting enzyme activity [32,34,35]. The active groups of phenolic compounds (generally phenolic hydroxyl groups) can donate hydrogen atoms (hydrogen atom transfer) or electrons (single electron transfer) to free radicals to stop the free radical chain reaction, and the phenolic compounds themselves transform into stable aryloxyl radicals (ArO•) [36,37]; the antioxidant mechanism is shown in the Figure 2E.

Therefore, yeast fermentation can effectively enhance the antioxidant activity and α-glucosidase inhibition rate of whole Calamondin puree, which is an effective way of processing whole Calamondin.

### 2.8. Characteristic Metabolites and Metabolic Pathway Prediction

Based on the changes in the sugars, organic acids, free amino acids, phenolic compounds, limonoids, and volatile compounds, the characteristic metabolites before and after fermentation were screened using the fold change (FC) of the metabolites. Metabolites with FC ≥ 1.5 or ≤ 0.5 and *p* < 0.05 are usually defined as differential metabolites, and the results are presented in Supplementary Table S1. Compared to those in the control, *P. terricola* QJJY1, *H. opuntiae* QJJY14, *H. opuntiae* QJJY15, and *P. terricola* QJJY17 produced 53, 59, 58, and 54 differential metabolites, respectively, and 42 common different metabolites were found in the samples fermented with each yeast.

An enrichment analysis of the metabolic pathways was conducted on the Majorbio Cloud platform (https://cloud.majorbio.com (accessed on 19 August 2024)). After log_10_ treatment of the number of differential metabolites contained in each pathway, a bubble map of the abundance of differential metabolite pathways was drawn (Supplementary Figure S3). Larger bubbles in the figure indicate higher pathway abundance. There were 29 common pathways in the fermentation process of the four yeast strains, and the main pathways were chemical structure transformation maps, biosynthesis of other secondary metabolites, cancer overview, digestive system, carbohydrate metabolism, and amino acid metabolism. The specific metabolic pathways of *P. terricola* QJJY1 and *P. terricola* QJJY17 were endocrine and metabolic diseases, while those of *H. opuntiae* QJJY14 and *H. opuntiae* QJJY15 were environmental adaptations.

**Figure 3:**
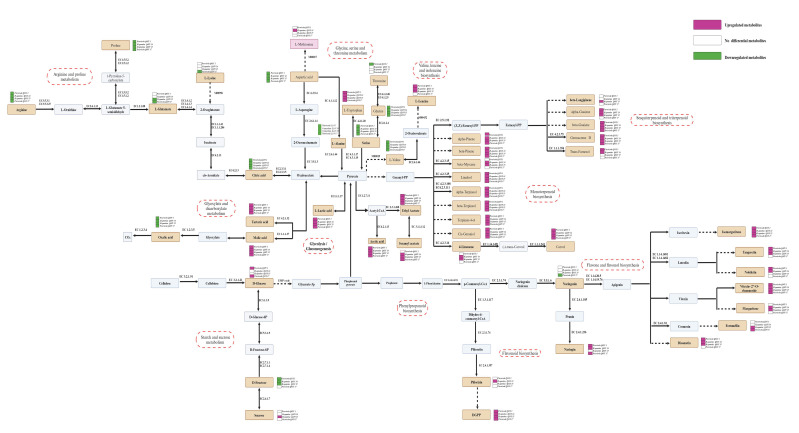
Prediction of the metabolic pathways of characteristic differential metabolites. The red scale diagram represents the upregulated metabolites; the white scale diagram represents the nondifferential metabolites; the green scale diagram represents the downregulated metabolites; the earthy yellow blocks represent characteristic differential metabolites; the red dotted blocks represents differential metabolic pathways.

The metabolic network was reconstructed based on KEGG and MetaCyc data, and the metabolic pathways of the characteristic differential metabolites were predicted (Figure 3). Cellulose and sucrose were converted into glucose through cellulase (EC3.2.1.91) and β-glucosidase (EC3.2.1.21) via yeast fermentation, which may have been the reason for the increase in the glucose content in the puree following *P. terricola* fermentation. Then, glucose was metabolized to glycerate-3p through the tricarboxylic acid cycle (EMP) and subsequently converted to phosphoenol pyruvate. Phosphoenol pyruvate was converted into prephenate for the metabolism of phenolic compounds or converted into pyruvate for the metabolism of organic acids, free amino acids, and volatile compounds [20].

Pyruvate was converted into lactic acid by lactic acid dehydrogenase (EC1.1.1.27) and 2-oxoacid oxidoreductase (EC1.2.7.11) or converted into acetic acid by acetate thiokinase (EC6.2.1.13). This may have been the reason for the increased lactic and acetic acid contents in the samples after fermentation. The citric acid content in Calamondin is very high, which is the main reason for its sour taste. Under the catalysis of citrate (Si)-synthase (EC2.3.3.1), methylcitrate synthetase (EC2.3.3.5), malate dehydrogenase (EC1.1.1.37), glyoxylate oxidase (EC1.2.3.5), and citric acid were converted into oxaloacetate, malic acid, and oxalic acid successively and finally degraded into CO_2_ by oxalate oxidase (EC1.2.3.4). This may have been a reason that fermentation reduced the citric acid content and improved the taste of the whole puree.

The main metabolic pathways related to differential amino acids were arginine and proline metabolism; glycine, serine, and threonine metabolism; and valine, leucine, and isoleucine biosynthesis. Pyruvate was transformed into L-leucine by the valine, leucine, and isoleucine biosynthesis pathway, which induced an increase in the L-leucine content in the fermented samples (except for *P. terricola* QJJY17). L-tryptophan can be transformed from threonine, glycine, and serine under the catalysis of chondroitin AC lyase (EC4.2.2.5), serine aldolase (EC 2.1.2.1), and glycerol dehydratase (EC4.2.1.30), which induce an increase in the L-tryptophan content and decreases in the threonine, glycine, and serine contents in the fermented samples.

There are two main ways to synthesize differential volatile compounds. Pyruvate is converted into acetyl-CoA by 2-oxoacid oxidoreductase (EC1.2.7.11) to synthesize esters (ethyl acetate and isoamyl acetate) or converted into geranyl-PP to synthesize terpene hydrocarbons, higher alcohols, and ketones by the sesquiterpenoid and triterpenoid biosynthesis or monoterpenoid biosynthesis pathways. Judging from the types and contents of volatile compounds, the isolated yeasts in this study may have been more inclined toward terpene hydrocarbon biosynthesis, and *H. opuntiae* had more abundant pathways in the synthesis of terpene hydrocarbons than *P. terricola*.

In this study, the synthesis of differential phenolic metabolites occurred through the phenylpropane metabolic branch. The results are consistent with those reported by Shen et al. [20]. Phenylalanine was converted to p-coumaroyl-CoA by trans-cinnamate 4-monooxygenase (EC1.14.14.91) and then transformed into dihydrochalcones, such as phlorizin and DGPP, by hydroxycinnamoyl-CoA reductase (EC1.3.1.117) and chalcone synthase (EC2.3.1.74). Phenylalanine was also converted to chalcones by chalcone synthase (EC2.3.1.74), then isomerized to flavanone aglycones (naringenin and apigenin) by chalcone isomerase (EC5.5.1.6), and finally converted to flavonoid glycosides (naringin, isomargaritene, tangeretin, vitexin-2″-orhamnoside, margaritene, fortunellin, and diosmetin) by glycosidic transferases.

## 3. Material and Methods

### 3.1. Samples and Chemical Reagents

Calamondin was purchased from Hainan Qingjingju Food Technology Co., Ltd. (Haikou, China). In all, 7 organic acid standards (HPLC ≥ 98%) (oxalic acid, tartaric acid, malic acid, lactic acid, acetic acid, citric acid, and succinic acid), 18 natural amino acid standards (B29396), 13 phenolic standards (HPLC ≥ 98%) (gallic acid, chlorogenic acid, gentisuric acid, vicenin-2, eriocitrin, naringin, diosmetin, margaritene, hesperidin, phlorizin, naringenin, nobiletin, tangeretin, and 7-hydroxycoumarin), and 3 limonin standards (HPLC ≥ 98%) (limonin glucoside, limonin, nomilin) were purchased from Yuanye Bio-Technology Co., Ltd. (Shanghai, China). The other 5 phenolic standards (HPLC ≥ 98%) (vitexin-2″-O-rhamnoside, DGPP, isomargaritene, fortunellin, and margaritene) were purchased from Wuhan Tianzhi Biotechnology Co., Ltd. (Wuhan, China) α-Glucosidase (derived from yeast S10050-100U), p-nitrophenyl-α-D-glucopyranoside (PNPG), neocuproine (T24013), 1,1-diphenyl-2-picrylhydrazyl (DPPH), 2,2-azino-bis (3-ethylbenzothiazoline-6-sulfonic acid) diammonium salt (ABTS), phenyl isothiocyanate (PITC), trimethylamine, and other analytical-grade chemical reagents were purchased from Yuanye Bio-Technology Co., Ltd. (Shanghai, China).

### 3.2. Yeast Isolation and Identification

The Calamondin puree was obtained from mature and intact fruit by crushing the whole fruit and grinding it through a colloid mill (DMM-40, Shanghai Qinshuo Chemical Machinery Equipment Co., Ltd., Shanghai, China) 3 times. Five kilograms of whole fruit puree was put into an aseptic fermentation tank for natural fermentation for 10 days to obtain samples. The samples were first diluted using the serial dilution method. The diluent (100 μL) was plated on YPD solid medium, which contained peptone (1% (*w*/*v*)), glucose (2% (*w*/*v*)), yeast powder (2% (*w*/*v*)), and agar (2% (*w*/*v*)), and then the mixtures were cultured at 28 °C for 3 days. The purification of yeast colonies was based on the morphology present on the plates.

The pure yeasts were inoculated in YPD broth (1% (*w*/*v*) peptone, 2% (*w*/*v*) glucose, and 2% (*w*/*v*) yeast powder) and incubated for 48 h at 28 °C. Yeast cells were collected via centrifugation (4 °C, 1800× *g*, 10 min) after a period of incubation. The collected yeast cells were washed twice with a sterile 0.9% (*w*/*v*) NaCl solution and made into the inoculum (10^7^ CFU/mL) with the same solution. The inoculum (0.5 mL) was added to the pasteurized (115 °C, 15 min) whole Calamondin puree (50 mL). After incubation at 27 ± 1 °C for 5 days, the number of viable yeast cells was measured using the plate counting method, and the yeast that could survive in the whole fruit puree was screened.

The isolated strains with strong viability were submitted to morphological analysis and identification based on an internal transcribed spacer (ITS) [38]. The polymerase chain reaction (PCR) products of each yeast were sequenced by Pengxiang Bio (Pengxiang Biology Co., Ltd., Qingdao, China), primer sequence: ITS-F CCGTGTTTCAAGACGGG; ITS-R CTTGGTCATTTAGAGGAAGTAA. The sequencing results were compared to the NCBI GenBank data library using a basic local alignment search tool (BLAST). Phylogenetic trees were constructed using the neighbor-joining method in MEGA 7.0.

### 3.3. Use of Isolated Yeast Stains in Whole Fruit Puree Fermentation

*Pichia terricola* QJJY1, *Hanseniaspora opuntiae* QJJY14, *Hanseniaspora opuntiae* QJJY15, and *Pichia terricola* QJJY17 were inoculated in YPD broth (compositions as shown in Section 2.2) and incubated for 48 h at 28 °C. Then, the yeast cells were collected via centrifugation (4 °C, 1800× *g*, 10 min), washed twice with a sterile 0.9% (*w*/*v*) NaCl solution, and made into inoculum (10^8^ CFU/mL) using the same solution. Then, 3.75 mL of the inoculum was added to 150 mL of pasteurized whole Calamondin puree (115 °C, 15 min) and then incubated at 27 ± 1 °C for 20 days. An equal volume of sterile 0.9% (*w*/*v*) NaCl solution, instead of inoculum, was used as a control. After fermentation, the samples were divided into two parts, one of which was directly used to analyze the pH value, viable yeast count, volatile compounds, and sensory evaluation. The other part was made into a freeze-dried powder via lyophilization to determine organic acids, sugars, amino acids, phenols, limonins, and functional activities. All of the fermentation experiments were repeated three times.

### 3.4. Determination of Sugars, Organic Acids, and Free Amino Acids

#### 3.4.1. Preparation of the Sugar, Organic Acid, and Amino Acid Extracts

The fermented freeze-dried samples (1.0 g) were extracted with distilled water (50.0 mL) via ultrasonic extraction (360 W, 50 °C) for 30 min. The extracts were centrifuged (10 min, 5000 r/min) and filtered using a 0.22 μm hydrophilic polytetrafluoroethylene (PTFE) membrane filter (Anpel Laboratory Technologies Inc., Shanghai, China). The filtrates were used to determine sugars, organic acids, and amino acids.

#### 3.4.2. Determination of Sugars

The measurement of sugars followed the methodology described by Georgelis et al. [39] with slight modifications. The sugars (sucrose, fructose, and glucose) were determined using high-performance liquid chromatography (HPLC) (Ultimate 3000, Thermo Fisher Scientific, Waltham, MA, USA) coupled with a refractive index detector (RID) and an Anthena NH2-RP column (4.6 × 250 mm, 5 μm) under equal degree elution conditions. The RID detector and column temperatures were set at 40 and 35 °C, respectively. The flow rate and injection volume were set at 1 mL/min and 10 μL, respectively. Acetonitrile (75%, *v*/*v*) and water (25%, *v*/*v*) mixtures were used as the mobile phase. The standard curve was drawn with different concentrations of standard substance as a horizontal coordinate and peak area as a vertical coordinate to calculate the sugar content in the sample. The results are expressed as grams per kilogram of the freeze-dried sample.

#### 3.4.3. Determination of Organic Acids

The measurement of organic acids followed the method described by Zeppa et al. [40] with slight modifications. Seven kinds of organic acids were determined using HPLC (Ultimate 3000, Thermo Fisher Scientific, Waltham, MA, USA) coupled with an ultraviolet absorption detector (UV) and a Thermo Acclaim 120 C18 column (4.6 × 250 mm, 5 μm) under equal degree elution conditions. The UV detector wavelength, column temperature, and injection volume were set at 210 nm, 30 °C, and 10 μL, respectively. The flow rate of the mobile phase was set at 0.8 mL/min, in which the mobile phases were the mixtures of methanol (2%, *v*/*v*) and 0.01 mol/L of potassium dihydrogen phosphate (98%, *v*/*v*) (pH 2.85). The results are expressed as grams per kilogram of the freeze-dried sample.

#### 3.4.4. Determination of Free Amino Acids

Eighteen kinds of free amino acids were determined using HPLC (Ultimate 3000, Thermo Fisher, Waltham, MA, USA) coupled with a diode array detector (DAD) and an Ultimate Amino Acid column (4.6 × 250 mm, 5 μm) under gradient elution conditions. The filtrate (400 μL), PITC acetonitrile solution (200 μL, 0.1 mol/L), triethylamine acetonitrile solution (200 μL 1 mol/L), and n-hexane (800 μL) were mixed and then left for 60 min to produce layers. The lower layer of the solution was filtered using a 0.22 μm organic nylon membrane filter and then analyzed using HPLC. The parameters of the liquid chromatography were as follows: column temperature set at 40 °C, wavelength set at 254 nm, flow rate set at 1.0 mL/min, and injection volume set at 10 μL. The mobile phases A and B consisted of sodium acetate (0.1 mol/L, pH 6.50) and anhydrous acetonitrile, respectively. The gradient elution procedure was set as follows: 0~4 min, 95% A; 4.01~31 min, 95%A → 67% A; 31.01~40 min, 67% A → 65% A; 40.01~45 min, 65% A → 10% A; 45.01~53 min, 10% A; 53.01~55 min, 10%A → 95% A; 55.01~67 min, 95% A. The results are expressed as grams per kilogram of the freeze-dried sample.

### 3.5. Determination of Phenolic Compounds and Limonoids

#### 3.5.1. Preparation of Phenolic Compound and Limonoid Extracts

The extraction of phenolic compounds and limonoids from fermented FWCP was performed using the method of Li et al. (2014) with appropriate modifications [41]. The lyophilized samples (0.2 g) and methanol (10 mL, 70% (*v*/*v*)) were mixed and added to a centrifuge tube (15 mL) and then treated using ultrasound (360 W, 50 °C) for 30 min. The treated mixtures were centrifuged (5000 r/min, 10 min) to obtain the supernatant, which was used for the determination of phenols and limonins after being filtered using a 0.22 μm organic nylon (PVDF) membrane filter.

#### 3.5.2. Determination of Phenolic Compounds and Limonins

The volatile compounds were determined using the method reported by Lou et al. (2014) with modifications [3]. Briefly, the phenolic compounds were determined using a Thermo Ultimate 3000 (Thermo Fisher Scientific, Waltham, MA, USA) coupled with a UV detector and a Thermo Acclaim 120 C18 column (4.6 × 250 mm, 5 μm), in which acetonitrile and formic acid (0.1% (*v*/*v*)), as well as water and formic acid (0.1% (*v*/*v*)), were used as mobile phases A and B, respectively. The column temperature, UV detector, and injection volume were set at 30 °C, 210 nm, and 10 μL, respectively. The gradient elution procedure was set as follows: 0–40 min, 5–25% A; 40–60 min, 25–100% A; 60–70 min, 100% A; 70–73 min, 100–5% A; 73–80 min, 5% A.

Phosphoric acid (3 mmol/L) and an acetonitrile solution were used as mobile phases A and B, respectively, to determine limonin glucoside, limonin, and nomilin. The column temperature, UV detector, and injection volume were set at 30 °C, 210 nm, and 10 μL. The gradient elution procedure was set as follows: 0–5 min, 85–77% A; 5–25 min, 77–74% A; 25–30 min, 74–60% A; 30–45 min, 60–54% A; 45–50 min, 54–85% A; 50–55 min, 85% A.

### 3.6. Determination of Volatile Compounds

The volatile compounds were determined using the method reported by Hwang et al. (2020) with modifications [42]. The sample (5.0 g) and dimethylheptane (20 μL, 139.6 ng/mL) used as the internal standard were added into a static headspace injection bottle, and then the volatile compounds of the sample were analyzed using a gas chromatography–mass spectrometry instrument (Thermo ISQ 7000, Thermo Fisher Scientific, Singapore) with a TG-5 SILMS capillary column (30 × 0.25 mm, 0.25 μm). The samples were incubated at 80 °C and 75 kPa for 30 min. The injection was carried out in split mode (split ratio: 50:1). The flow rate of the carrier gas nitrogen was set at 1.0 mL/min. The electron ionization source and transmission line temperatures were set at 300 and 270 °C, respectively. The ionization energy was set at 70 EV. The temperature of the column was heated up as follows: heated to 35 °C and maintained for 5 min; increased to 180 °C at 3 °C/min and held for 1 min; increased to 260 °C at 30 °C/min and held for 5 min. The qualitative determination of volatile compounds was performed by comparing the linear index of retention (LRI; determined by n-alkanes C7–C40) with the standard in the National Institute of Standards and Technology (NIST) library. The semiquantitative determination of volatile compounds was based on the peak area of the internal standard, and the results are expressed in micrograms per kilogram of the sample solution.

### 3.7. Evaluation of the Antioxidant Activity and A-Glucosidase Inhibition Rate

#### 3.7.1. DPPH (2,2-Diphenyl-1-picrylhydrazyl) Radical Scavenging Activity

The 2,2-diphenyl-1-picrylhydrazyl (DPPH) radical scavenging activity was determined using the method reported by Xia et al. (2020) [43] with modifications. The extracted samples were prepared as detailed in Section 3.5.1. The extracted sample (36 μL), methanol solution (174 μL, 70% (*v*/*v*)), and DPPH solution (40 μL, 1 mmol/L) were mixed and then loaded into a 96-well plate to react for 30 min in the dark at room temperature. The absorbance of the samples and control was measured at 517 nm. The DPPH radical scavenging activity was calculated according to the following formula:

$$\mathrm{DPPH}\;\mathrm{radical}\;\mathrm{scavenging}\;\mathrm{activity}\left(\%\right)=\frac{A_{C}-A_{s}}{A_{c}}\times 100\%$$

where Ac is the absorbance of the DPPH solution at 517 nm without adding the sample, and As is the absorbance of the DPPH solution at 517 nm after reaction with the sample.

#### 3.7.2. ABTS Radical Cation Scavenging Activity

The ABTS radical cation (ABTS^+^) scavenging ability was determined using the method reported by Fan et al. (2018) with appropriate modifications [44]. The ABTS^+^ solution was prepared by mixing ABTS^+^ (7 mmol/L) with a potassium persulfate solution (2.4 mmol/L) in the same volume and then reacting it at room temperature for 12–16 h in the dark. Before use, the freshly prepared ABTS^+^ solution was diluted with methanol to an absorbance of 0.7 ± 0.02 (734 nm). The ABTS^+^ diluent solution (150 μL); extract samples (9 μL), which was prepared according to the method in Section 3.5.1; and methanol (21 μL, 70% (*v*/*v*)) were mixed and then loaded into a 96-well plate for reacting for 10 min in the dark at room temperature. The absorption value was measured at 734 nm. The ABTS^+^ scavenging capacity was calculated according to the following formula:

$$\mathrm{ABTS}\;\mathrm{radical}\;\mathrm{scavenging}\;\mathrm{activity}\left(\%\right)=\frac{A_{C}-A_{s}}{A_{c}}\times 100\%$$

where Ac is the absorbance of the ABTS^+^ solution at 734 nm without adding the sample, and As is the absorbance of the ABTS^+^ solution at 734 nm after reaction with the sample.

#### 3.7.3. Cupric Reducing Antioxidant Capacity

The cupric reducing antioxidant capacity (CUPRAC) was determined according to a previously reported method [45]. The extracted samples were prepared as detailed in Section 3.5.1. The extracted sample (25 μL), CuCl_2_ (50 μL, 10 mmol/L), new cuprous reagent solution (50 μL, 7.5 mmol/L), ammonium acetate buffer (pH 7, 50 μL, 1 mol/L), and distilled water (30 μL) were mixed and then loaded into a 96-well enzyme-labeled plate. The mixtures were reacted for 30 min at room temperature, and their absorbance was determined at 450 nm. Those mixtures with 70% methanol were used as blanks. A standard solution of gallic acid (GA) with different concentrations (5, 10, 20, 30, and 40 μg/L) was used to measure the absorbance instead of the sample solution, and a standard curve of the absorbance and concentration of GA was drawn. The CUPRAC of the sample is expressed as micrograms of GA per gram of the freeze-dried sample.

#### 3.7.4. Determination of the α-Glucosidase Inhibition Rate

The α-glucosidase inhibition rate was determined using the method reported by Fu et al. (2022) [46]. The extracted samples were prepared as detailed in Section 3.5.1. The extracted sample (2 mL) was first dried in an evaporating vacuum at 50 °C (ZLS-2 vacuum centrifugal concentrator, Hesi Instrument Equipment, Changsha, China) and then redissolved using dimethyl sulfoxide (2 mL, 5% (*v*/*v*)) to determine the inhibition rate of α-glucosidase enzymes. Next, the redissolved solution (10 μL) and α-glucosidase (50 μL, 1.0 U/mL) were mixed and loaded into a 96-well plate, followed by incubation at 37 °C for 15 min. Then, PNPG (50 μL, 5 mmol/L) was added to the 96-well plate and incubated at 37 °C for 15 min. After this, the sodium carbonate solution (150 μL, 0.5 mol/L) was added to terminate the reaction, and the absorbance of the mixture was measured at 415 nm. The α-glucosidase activity is expressed as p-nitrophenol released from PNPG.

$$\mathrm{GIR}=\frac{{(\mathrm{ABS}}_{\mathrm{Control}}-{\mathrm{ABS}}_{\mathrm{Blank}\;\mathrm{Control}})-\left({\mathrm{ABS}}_{\mathrm{Sample}}-{\mathrm{ABS}}_{\mathrm{Blank}\;\mathrm{Control}}\right)}{\left({\mathrm{ABS}}_{\mathrm{Contral}}-{\mathrm{ABS}}_{\mathrm{Blank}\;\mathrm{Control}}\right)}\times 100\%$$

Here, GIR is the α-glucosidase inhibition rate (%); ABS_Control_ is the absorbance of the reaction between the enzyme and substrate; ABS_Blank Control_ is the absorbance of the substrate without the enzyme; ABS_Sample_ is the absorbance of the reaction between the sample, enzyme, and substrate; and ABS_Blank Sample_ is the absorbance of the reaction between the sample and substrate.

### 3.8. Sensory Assessment

Samples were pasteurized before sensory assessment. Unfermented and fermented samples using *P. terricola* QJJY1, *H. opuntiae* QJJY14, *H. opuntiae* QJJY15, and *P. terricola* QJJY17 were evaluated through sensory analysis. The samples (10 mL) were poured into a glass and randomly delivered to the judges. Grades (0–5, where 0 is minimal, 5 is maximal) were assigned based on color, flavor, taste, and organization status. The detailed evaluation criteria are shown in Supplementary Table S2.

### 3.9. Data Analysis

Data were analyzed via analysis of variance (ANOVA) and Dunn’s test (*p* < 0.05) using SPSS (IBM SPSS Statistics 23). Principal component analysis (PCA) was performed based on the data for the volatile compounds, basic physicochemical composition (organic acids and sugars), nutrient substance (phenolic compounds and limonoids), and amino acids to differentiate among *P. terricola* QJJY1, *H. opuntiae* QJJY14, *H. opuntiae* QJJY15, and *P. terricola* QJJY17 using Origin 2019b. Enrichment analysis of metabolic pathways was carried out using the Majorbio Cloud platform (https://cloud.majorbio.com (accessed on 16 August 2024)). The metabolite pathway prediction was designed using the KEGG pathway and MetaCyc databases. All experiments were performed in triplicate. The data are presented as mean values and standard deviations.

## 4. Conclusions

In this study, whole Calamondin puree was fermented with yeasts isolated from naturally fermented Calamondin. The whole Calamondin puree was suitable for yeast fermentation, and the isolated yeasts (*P. terricola* QJJY1, *H. opuntiae* QJJY14, *H. opuntiae* QJJY15, and *P. terricola* QJJY17) had good survival ability (10^8^ CFU/mL) and high metabolic activity in the whole-fruit puree during fermentation (20 d). Fermentation with the selected yeasts decreased the sour taste by metabolizing citric acid (decreasing by 73.28–80.51%) and enhanced the aroma by promoting the release of volatile compounds (increasing by 34.40–65.30%) and the synthesis of new flavor compounds (isoamyl acetate) in the whole Calamondin puree, which significantly improved the puree’s sensory scores. Yeast fermentation also promoted the release of bound phenols, resulting in a higher phenolic content (DGPP, vitexin-2″-o-rhamnoside, isomargaritene) in the fruit puree, which significantly enhanced the DPPH radical scavenging activity (74.22–103.52%), ABTS radical cation scavenging activity (140.15–158.67%), cupric reducing antioxidant capacity (94.17–180.58%), and the inhibition activity of α-glucosidase (24.77–28.42%). The results provide strong evidence that yeast fermentation is a promising bioprocessing technology for improving the sensory quality and nutrient content of fruits.

## Figures and Tables

**Table 1:** Concentrations of free amino acid in nonfermented (control) and fermented whole Calamondin puree (20 days) using *P. terricola* QJJY1, *H. opuntiae* QJJY14, *H. opuntiae* QJJY15, and *P. terricola* QJJY17.

Free Amino Acid (mg/g)	Control	*P. terricola* QJJY1	*H. opuntiae* QJJY14	*H. opuntiae* QJJY15	*P. terricola* QJJY17
Asp	6.41 ± 0.20 c	2.30 ± 0.07 b	1.43 ± 0.09 a	1.17 ± 0.19 a	1.36 ± 0.30 a
Glu	1.74 ± 0.13 d	1.27 ± 0.03 c	0.96 ± 0.03 b	0.59 ± 0.04 a	0.63 ± 0.12 a
Ser	1.80 ± 0.04 d	0.26 ± 0.02 c	0.15 ± 0.07 b	0.06 ± 0.00 a	0.10 ± 0.04 ab
Gly	0.22 ± 0.02 b	0.09 ± 0.01 a	0.09 ± 0.03 a	0.07 ± 0.01 a	0.08 ± 0.01 a
His	0.33 ± 0.05 ab	0.40 ± 0.02 bc	0.31 ± 0.01 a	0.25 ± 0.08 a	0.42 ± 0.02 c
Arg	0.33 ± 0.02 b	0.06 ± 0.01 a	0.08 ± 0.02 a	0.08 ± 0.02 a	0.06 ± 0.02 a
Thr	0.32 ± 0.02 bc	0.06 ± 0.01 a	0.33 ± 0.01 c	0.29 ± 0.03 b	0.32 ± 0.03 bc
Ala	0.87 ± 0.02 c	0.43 ± 0.04 b	0.21 ± 0.01 a	0.23 ± 0.05 a	0.27 ± 0.02 a
Pro	1.96 ± 0.05 c	0.32 ± 0.06 b	0.19 ± 0.01 a	0.20 ± 0.02 a	0.34 ± 0.09 b
Tyr	0.18 ± 0.00 b	0.07 ± 0.01 a	0.09 ± 0.04 a	0.05 ± 0.03 a	0.08 ± 0.01 a
Val	0.28 ± 0.01 b	0.07 ± 0.01 a	0.11 ± 0.06 a	0.06 ± 0.01 a	0.06 ± 0.02 a
Met	0.03 ± 0.00 a	0.04 ± 0.01 a	0.06 ± 0.02 a	0.04 ± 0.01 a	0.04 ± 0.01 a
Cys	0.36 ± 0.00 a	0.38 ± 0.01 ab	0.38 ± 0.01 ab	0.38 ± 0.01 ab	0.39 ± 0.01 b
Ile	0.10 ± 0.00 a	0.08 ± 0.02 a	0.11 ± 0.03 a	0.09 ± 0.02 a	0.09 ± 0.02 a
Leu	0.09 ± 0.00 a	0.13 ± 0.01 abc	0.19 ± 0.06 c	0.15 ± 0.03 bc	0.12 ± 0.03 ab
Phe	0.59 ± 0.01 b	0.19 ± 0.00 a	0.20 ± 0.03 a	0.17 ± 0.03 a	0.18 ± 0.00 a
Trp	0.05 ± 0.00 a	0.15 ± 0.01 b	0.18 ± 0.00 b	0.16 ± 0.03 b	0.17 ± 0.02 b
Lys	0.29 ± 0.00 d	0.13 ± 0.01 a	0.25 ± 0.05 cd	0.19 ± 0.03 bc	0.14 ± 0.02 ab
Total amino acids	15.96 ± 0.30 d	6.44 ± 0.17 c	5.31 ± 0.39 b	4.24 ± 0.36 a	4.84 ± 0.52 ab

Note: Each value is expressed as the mean ± standard deviation (*n* = 3); control: nonfermented whole Calamondin puree; fermented whole Calamondin puree: *P. terricola* QJJY1, *H. opuntiae* QJJY14, *H. opuntiae* QJJY15, or *P. terricola* QJJY17. Means values with different letters in the same line indicate a significant difference (*p* < 0.05).

**Table 2:** Concentrations of volatile compounds in nonfermented (control) and fermented whole Calamondin puree (20 days) using *P. terricola* QJJY1, *H. opuntiae* QJJY14, *H. opuntiae* QJJY15, and *P. terricola* QJJY17.

No.	Volatile Compounds	LRI	CAS	μg/kg					Odor Description
				Control	*P. terricola* QJJY1	*H. opuntiae* QJJY14	*H. opuntiae* QJJY15	*P. terricola* QJJY17	
	Higher alcohols			3668.57 ± 296.87 a	7164.43 ± 893.14 b	7423.03 ± 754.98 b	7699.94 ± 1629.31 b	6762.57 ± 230.07 b	
1	2-methyl-3-Buten-2-ol ^A^	659	115-18-4	12.93 ± 1.23 a	149.22 ± 29.07 b	181.11 ± 12.3 bc	224.21 ± 52.41 c	193.93 ± 7.28 bc	Herb
2	Linalool	1098	78-70-6	132.14 ± 21.57 a	985.6 ± 204.04 b	963.46 ± 34.27 b	1068.83 ± 262.64 b	865.23 ± 38.23 b	Coriander, floral, lemon, rose
3	β-Terpineol ^A^	1146	138-87-4	237.2 ± 19.61 a	577.04 ± 63.63 b	581.2 ± 90.47 b	597.44 ± 127.67 b	528.45 ± 15.64 b	Coriander
4	1-Nonanol	1171	143-08-8	0.08 ± 0.14 a	5.58 ± 5.67 a	n.d.	n.d.	n.d.	Fat, floral, green, oil
5	Terpinen-4-ol	1176	562-74-3	188.48 ± 21.91 a	667.28 ± 104.09 b	686.58 ± 50.95 b	729.83 ± 162.67 b	629.52 ± 26.04 b	Earth, must, nutmeg, wood
6	α-Terpineol	1190	98-55-5	2982.44 ± 220.97 a	4447.84 ± 434.86 b	4694.05 ± 537.97 b	4784.78 ± 979.69 b	4304.11 ± 122.17 b	Anise, fresh, mint, oil
7	Ageratriol	1215	38022-97-8	n.d.	2.81 ± 4.86 ab	8.42 ± 0.55 b	6.78 ± 2.49 ab	5.93 ± 6.36 ab	n.a.
8	cis-Geraniol ^A^	1250	106-25-2	n.d.	24.36 ± 7.4 b	16.87 ± 13.94 ab	2.49 ± 4.32 a	9.76 ± 16.91 ab	Sweet
9	trans-Farnesol ^A^	1347	106-28-5	8.22 ± 0.72 a	26.8 ± 5.83 b	13.98 ± 5.24 a	7.8 ± 1.29 a	8.98 ± 2.28 a	Muguet
10	2-Naphthalenemethanol	1646	1592-38-7	107.07 ± 10.99 a	277.9 ± 47.29 b	277.37 ± 38.97 b	277.77 ± 44.66 b	216.67 ± 14.31 b	n.a.
	Aldehydes			207.97 ± 38.51 b	21.55 ± 14.35 a	0 ± 0 a	0 ± 0 a	0 ± 0 a	
11	Nonanal	1102	124-19-6	100.01 ± 21.96 b	6.49 ± 1.83 a	n.d.	n.d.	n.d.	Fat, floral, green, lemon
12	Decanal	1203	112-31-2	105.26 ± 16.52 b	15.07 ± 13.05 a	n.d.	n.d.	n.d.	Floral, fried, orange peel, penetrating, tallow
13	(E)-2-Decenal ^A^	1259	3913-81-3	2.7 ± 0.02 b	n.d.	n.d.	n.d.	n.d.	Tallow
	Esters			395.91 ± 33.4 a	649.12 ± 109.28 b	357.93 ± 63.73 a	392.78 ± 117.57 a	239.12 ± 107.8 a	
14	Ethyl acetate	668	141-78-6	0.12 ± 0.05 a	1.11 ± 0.53 b	0.71 ± 0.45 ab	0.51 ± 0.13 ab	0.33 ± 0.1 a	Aromatic, brandy, grape
15	Isoamyl acetate^A^	726	123-92-2	n.d.	n.d.	213.84 ± 37.86 ab	280.86 ± 67.85 c	130.53 ± 100.94 a	Banana
16	1-Octanol acetate ^B^	1209	112-14-1	38.25 ± 5.34 a	34.23 ± 9.7 a	n.d.	n.d.	n.d.	Green, earthy, mushroom
17	Geranyl propionate	1356	105-90-8	44.1 ± 2.81 b	92.75 ± 13.91 c	35.96 ± 13.9 ab	19.38 ± 8.78 a	19.53 ± 4.7 a	Floral
18	Geranyl isovalerate	1375	109-20-6	313.43 ± 25.2 b	521.04 ± 86.34 c	107.41 ± 36.62 a	92.04 ± 43.79 a	88.73 ± 36.7 a	Apple, fruit, rose
	Terpene hydrocarbons			339,719.56 ± 79,433.24 a	455,810.93 ± 72,571.69 ab	512,905.53 ± 48,108.58 ab	560,466.59 ± 13,6886.41 b	465,057.92 ± 50,295.93 ab	
19	α-Pinene	929	80-56-8	2138.49 ± 599.44 a	3508.87 ± 590.55 b	4207.59 ± 341.72 b	4716.36 ± 1188.87 b	3856.71 ± 450.9 b	Cedarwood, pine, sharp
20	Camphene	944	79-92-5	48.13 ± 12.64 a	32.18 ± 7.87 a	39.18 ± 4.25 a	44.52 ± 9.67 a	37.19 ± 5.21 a	Camphor, mothball, oil, warm
21	β-Phellandrene ^A^	969	555-10-2	71.35 ± 21.65 a	66.34 ± 11.75 a	75.82 ± 16.92 a	79.31 ± 16.6 a	72.81 ± 6.11 a	Mint, turpentine
22	β-Pinene	972	127-91-3	168.06 ± 51.24 a	341.76 ± 66.02 b	410.66 ± 19.49 b	440.61 ± 124.2 b	356.7 ± 37.99 b	Pine, polish, wood
23	β-Myrcene	989	123-35-3	6148.43 ± 1616.86 a	8312.27 ± 1374.4 ab	9640.49 ± 986.98 b	10650.3 ± 2643.51 b	8680.54 ± 1032.66 ab	Balsamic, fruit, geranium, herb, must
24	α-Terpinen	1014	99-86-5	423.28 ± 104.83 a	435.78 ± 67.46 a	387.54 ± 66.98 a	408.52 ± 84.93 a	341.62 ± 45.4 a	Lemon
25	1,3,8-p-Menthatriene ^A^	1021	18368-95-1	68.46 ± 17.58 a	40.47 ± 39.58 a	64.13 ± 12.75 a	69.81 ± 22.56 a	52.59 ± 2.96 a	Turpentine
26	D-Limonene	1030	5989-27-5	329,499.45 ± 76,769.22 a	441,446.1 ± 70181.22 ab	496,630.38 ± 46,441.31 b	542,599.63 ± 132,418.24 b	450,571.98 ± 48,612.59 ab	Citrus, mint
27	3-Carene	1046	13466-78-9	70.99 ± 19.34 a	99.55 ± 21.21 a	94.91 ± 13.89 a	102.69 ± 26.78 a	79.88 ± 10.91 a	Lemon
28	γ-Terpinene	1056	99-85-4	221.98 ± 52.59 a	283.46 ± 44.08 a	290.35 ± 36.6 a	301.85 ± 72.18 a	245.66 ± 24.92 a	Bitter, citrus
29	3,7-dimethyl-1-Octene ^B^	1072	4984-01-4	13.91 ± 1.89 a	39.23 ± 11.48 b	50.94 ± 9.27 b	55.14 ± 15.47 b	49.61 ± 2.67 b	Woody, piney, herbaceous
30	Terpinolene	1082	586-62-9	615.94 ± 133.24 b	534.6 ± 73.55 ab	490.57 ± 93.1 ab	488.73 ± 93.78 ab	402.34 ± 36.95 a	Pine
31	2-ethenyl-1,1-dimethyl-3-methylene- Cyclohexane	1112	95452-08-7	7.77 ± 1.13 ab	4.97 ± 5.82 a	12.7 ± 2.33 b	9.17 ± 3.52 ab	6.42 ± 2.4 ab	n.a.
32	1,1-bis(dodecyloxy-Hexadecane	1407	56554-64-4	14.84 ± 1.29 b	29.9 ± 6.18 c	2.94 ± 5.09 a	n.d.	n.d.	n.a.
33	β-Longipinene	1412	39703-25-8	4.08 ± 0.49 a	2.88 ± 3.53 a	5.77 ± 5 a	8.52 ± 7.8 a	n.d.	n.a.
34	Germacrene D	1474	24150-39-8	125.84 ± 20.49 a	468.72 ± 87.5 c	339.87 ± 49.21 b	331.87 ± 100.4 b	195.26 ± 60.46 a	Floral, soapy, green
35	α-Guaiene	1482	53863-54-0	47.37 ± 6.33 a	94.27 ± 19.39 b	79.19 ± 13.41 b	80.67 ± 24.17 b	47.74 ± 13.76 a	n.a.
36	Alloaromadendrene ^A^	1489	25246-27-9	9.57 ± 0.57 a	31.49 ± 16.16 b	26.38 ± 11.19 ab	29.73 ± 8.88 b	20.62 ± 5.89 ab	Woody
37	β-Acorenol	1542	28400-11-5	6.45 ± 0.07 ab	n.d.	17.74 ± 4.75 c	17.08 ± 6.63 c	12.71 ± 1.07 bc	n.a.
38	β-Guaiene ^A^	1614	88-84-6	15.18 ± 0.12 a	41.07 ± 10.02 b	38.38 ± 10.38 b	32.07 ± 11.48 b	27.55 ± 3.17 ab	Woody, spice
	Ketones			10.79 ± 1.12 a	44.99 ± 8.25 b	56.86 ± 4.99 b	60.28 ± 18.22 b	43.82 ± 2.54 b	
39	Carvol	1239	2244-16-8	10.79 ± 1.12 a	44.99 ± 8.25 b	56.86 ± 4.99 b	60.28 ± 18.22 b	43.82 ± 2.54 b	Basil, bitter, caraway, fennel, mint

Each value is expressed as the mean ± SD (*n* = 3). Mean values with different letters in the same line indicate a significant difference (*p* < 0.05). n.d.: not detected; n.d.: not available. The addition of an “A” in the top right corner of a volatile substance indicates that its aroma descriptor was derived from http://www.flavornet.org/index.html (accessed on 17 July 2024). The addition of a “B” in the top right corner of a volatile substance indicates that its aroma descriptor was derived from http://www.thegoodscentscompany.com/index.html (accessed on 17 July 2024). Odor descriptors for unlabeled volatiles were derived from https://www.femaflavor.org/flavor-library (accessed on 17 July 2024).

**Table 3:** Concentrations of phenolic compounds and limonoids in nonfermented (control) and fermented whole Calamondin puree (20 days) using *P. terricola* QJJY1, *H. opuntiae* QJJY14, *H. opuntiae* QJJY15, and *P. terricola* QJJY17.

No.	Physicochemical Parameter (mg/g)	Control	*P. terricola* QJJY1	*H. opuntiae* QJJY14	*H. opuntiae* QJJY15	*P. terricola* QJJY17
	Total polyphenols	15.03 ± 0.26 a	22.97 ± 0.94 b	22.75 ± 0.75 b	22.73 ± 0.67 b	22.15 ± 0.26 b
	Phenolic acids					
1	Gallic acid	0.09 ± 0.02 a	0.09 ± 0.01 a	0.07 ± 0.02 a	0.07 ± 0.01 a	0.14 ± 0.02 b
2	Chlorogenic acid	0.28 ± 0.04 a	0.29 ± 0.04 a	0.32 ± 0.05 a	0.32 ± 0.03 a	0.30 ± 0.05 a
3	Gentisuric acid	0.09 ± 0.05 a	0.12 ± 0.07 a	0.07 ± 0.02 a	0.07 ± 0.02 a	0.07 ± 0.01 a
	Flavone glycosides					
4	Vicenin-2	1.13 ± 0.17 a	1.47 ± 0.04 b	1.53 ± 0.08 b	1.44 ± 0.05 b	1.41 ± 0.02 b
5	Vitexin-2″-O-rhamnoside	0.68 ± 0.06 a	1.42 ± 0.04 c	1.32 ± 0.06 b	1.29 ± 0.05 b	1.35 ± 0.02 bc
6	Eriocitrin	1.42 ± 0.02 a	1.94 ± 0.19 b	2.00 ± 0.1 b	1.85 ± 0.12 b	1.82 ± 0.05 b
7	DGPP	8.02 ± 0.113 a	12.58 ± 0.34 c	12.43 ± 0.20 bc	12.44 ± 0.19 bc	12.15 ± 0.12 b
8	Naringin	0.13 ± 0.03 a	0.22 ± 0.04 c	0.20 ± 0.04 bc	0.15 ± 0.03 ab	0.22 ± 0.02 c
9	Diosmetin	0.14 ± 0.02 a	0.32 ± 0.07 c	0.29 ± 0.03 c	0.25 ± 0.04 bc	0.19 ± 0.06 ab
10	Margaritene	0.17 ± 0.05 a	0.22 ± 0.05 a	0.28 ± 0.13 ab	0.39 ± 0.08 b	0.23 ± 0.03 a
11	Hesperiden	0.82 ± 0.03 a	1.17 ± 0.19 b	1.09 ± 0.13 b	1.19 ± 0.06 b	1.19 ± 0.05 b
12	Phlorizin	0.33 ± 0.05 a	0.44 ± 0.11 a	0.62 ± 0.07 b	0.48 ± 0.08 ab	0.47 ± 0.09 ab
13	Isomargaritene	0.17 ± 0.03 a	0.39 ± 0.04 c	0.31 ± 0.01 b	0.29 ± 0.06 b	0.26 ± 0.03 b
14	Fortunellin	0.27 ± 0.03 a	0.34 ± 0.15 a	0.35 ± 0.05 a	0.69 ± 0.09 b	0.61 ± 0.02 b
	Aglycons					
15	Naringenin	0.09 ± 0.01 b	0.07 ± 0.04 ab	0.06 ± 0.00 ab	0.05 ± 0.01 b	0.04 ± 0.00 b
16	Nobiletin	0.56 ± 0.01 a	0.91 ± 0.04 d	0.84 ± 0.03 c	0.82 ± 0.02 bc	0.78 ± 0.01 b
17	Tangeretin	0.56 ± 0.00 a	0.86 ± 0.06 b	0.85 ± 0.02 b	0.84 ± 0.02 b	0.81 ± 0.02 b
18	7-Hydroxycoumarin	0.07 ± 0.01 a	0.1 ± 0.02 a	0.09 ± 0.01 a	0.11 ± 0.03 a	0.08 ± 0.00 a
	Total limonoids	2.89 ± 0.07 a	3.61 ± 0.08 bc	3.61 ± 0.04 bc	3.50 ± 0.24 b	3.76 ± 0.10 c
19	Limonin glucoside	1.88 ± 0.04 a	2.18 ± 0.05 b	2.19 ± 0.05 b	2.08 ± 0.10 b	2.16 ± 0.08 b
20	Limonin	0.97 ± 0.02 a	1.35 ± 0.10 b	1.34 ± 0.01 b	1.33 ± 0.20 b	1.52 ± 0.04 b
21	Nomilin	0.04 ± 0.00 a	0.08 ± 0.01 b	0.08 ± 0.00 b	0.09 ± 0.02 b	0.07 ± 0.00 b

Note: Each value is expressed as the mean ± standard deviation (*n* = 3); control: nonfermented whole Calamondin puree; fermented whole Calamondin puree: *P. terricola* QJJY1, *H. opuntiae* QJJY14, *H. opuntiae* QJJY15, or *P. terricola* QJJY17. Mean values with different letters in the same line indicate a significant difference (*p* < 0.05).

## Data Availability

The original contributions presented in the study are included in the article/Supplementary Materials, further inquiries can be directed to the corresponding authors.

## Supplementary Materials

The supporting information can be downloaded at: https://www.mdpi.com/article/10.3390/ijms252211984/s1.
