# Supplementary material for: Effectively Enhancing the Physiological Activity and Sensory Quality of Whole Calamondin Puree via Yeast Fermentation
Source: Int J Mol Sci. 2024 Nov 7;25(22):11984. doi: 10.3390/ijms252211984 (PMC11593417; doi:10.3390/ijms252211984)
Supplement: Supplementary file 1 [file ijms-25-11984-s001.zip › ijms-3252629-supplementary.pdf]

Supplementary material

Supplementary Table S1. The characteristic differential metabolites (FC ≥ 1.5 or ≤ 0.5 and $p < 0.05$) in non-fermented (control) and fermented whole Calamondin puree (20 days) using *P. terricola QJJY1, H. opuntiae QJJY14, H. opuntiae QJJY15*, and *P. terricola QJJY17.*

No.	Metabolites	*P. terricola QJJY1*	*H. opuntiae QJJY14*	*H. opuntiae QJJY15*	*P. terricola QJJY17*
1	Oxalic acid	0.26	0.25	0.27	0.33
2	Tartaric acid	1.71	1.83	1.73	1.59
3	Malic acid	1.61	2.09	2.34	2.40
4	Lactic acid	2.96	2.75	2.90	2.86
5	Acetic acid	2.17	2.03	2.35	2.40
6	Citric acid	0.22	0.19	0.22	0.27
7	Glucose	4.15	N	N	3.10
8	Fructose	0.19	0.34	0.20	N
9	Sucrose	N	N	2.06	N
10	Asp	0.36	0.22	0.18	0.21
11	Glu	N	N	0.34	0.36
12	Ser	0.15	0.08	0.03	0.05
13	Gly	0.41	0.41	0.33	0.36
14	Arg	0.20	0.24	0.25	0.18
15	Thr	0.19	N	N	N
16	Ala	0.49	0.24	0.26	0.31
17	Pro	0.16	0.10	0.10	0.18
18	Tyr	0.38	0.48	0.29	0.42
19	Val	0.26	0.38	0.21	0.22
20	Met	N	1.83	N	N
21	Leu	1.54	2.22	1.78	N
22	Phe	0.33	0.34	0.28	0.31
23	Trp	2.97	3.59	3.27	3.45
24	Lys	N	N	N	0.49
25	Gallic acid	N	N	N	1.50
26	Vitexin-2"-o-rhamnoside	2.09	1.95	1.90	2.00
27	DGPP	1.57	1.55	1.55	1.51
28	Naringin	1.72	1.51	N	1.69
29	Diosmetin	2.29	2.10	1.80	N
30	Margaritene	N	1.70	2.36	N
31	Phlorizin	N	1.91	N	N
32	Isomargaritene	2.29	1.82	1.67	1.51
33	Fortunellin	N	N	2.59	2.30
34	Naringenin	N	N	N	0.41
35	Nobiletin	1.63	1.50	N	N
36	Tangeretin	1.55	1.53	1.51	N
37	Limonin	N	N	N	1.57
38	Nomilin	2.03	1.95	2.25	1.86
39	2-Methyl-3-buten-2-ol	11.54	14.00	17.34	14.99
40	Linalool	7.46	7.29	8.09	6.55
41	β-Terpineol	2.43	2.45	2.52	2.23
42	1-Nonanol	68.20	0.00	0.00	0.00
43	Terpinen-4-ol	3.54	3.64	3.87	3.34
44	α-Terpineol	N	1.57	1.60	N
45	Ageratriol	+∞	+∞	+∞	+∞
46	Cis-Geraniol	+∞	+∞	+∞	+∞
47	Trans-Farnesol	3.26	1.70	N	N
48	2-Naphthalenemethanol	2.60	2.59	2.59	2.02
49	Nonanal	0.06	0.00	0.00	0.00
50	Decanal	0.14	0.00	0.00	0.00
51	(E)-2-Decenal	0.00	0.00	0.00	0.00
52	Ethyl Acetate	9.04	5.81	4.16	2.72

53	Isoamyl acetate	N	+∞	+∞	+∞
54	1-Octanol acetate	N	0.00	0.00	0.00
55	Geranyl propionate	2.10	N	0.44	0.44
56	Geranyl isovalerate	1.66	0.34	0.29	0.28
57	α-Pinene	1.64	1.97	2.21	1.80
58	β-Pinene	2.03	2.44	2.62	2.12
59	β-Myrcene	N	1.57	1.73	N
60	D-Limonene	N	1.51	1.65	N
61	3,7-dimethyl-1-Octene	2.82	3.66	3.96	3.57
62	2-ethenyl-1,1-dimethyl-3-methylene-Cyclohexane	N	1.64	N	N
63	1,1-bis(dodecyloxy)-Hexadecane	2.02	0.20	0.00	0.00
64	β-Longipinene	N	N	2.09	0.00
65	Germacrene D	3.72	2.70	2.64	1.55
66	α-Guaiene	1.99	1.67	1.70	N
67	Alloaromadendrene	3.29	2.76	3.11	2.15
68	β-Acorenol	0.00	2.75	2.65	1.97
69	β-Guaiene	2.71	2.53	2.11	1.81
70	Carvol	4.17	5.27	5.59	4.06

"N" represents non-differential metabolites.

Supplementary Table S2. Sensory evaluation standards.

Items	Description	Score
Color/5	Bright yellow and uniform	4-5
	Light yellow or uneven color, light luster	2-4
	Dark yellow or uneven color, dull	0-2
Flavor/5	Typical rich Calamondin aroma and pure coordination of fermentation aromas	4-5
	Exhibits a Calamondin aroma but not rich, and a little fermentation aroma	2-4
	No Calamondin aroma or a bad aroma	0-2
Taste/5	Sour and bitter, refreshing on the mouth, good palatability	4-5
	A little bit tart or bitter, average flavor, average palatability	2-4
	Too sour or too bitter, poor taste	0-2
Organization status/5	Uniform, no stratification	3-5
	No uniform, stratification	0-3
Total/20		0-20

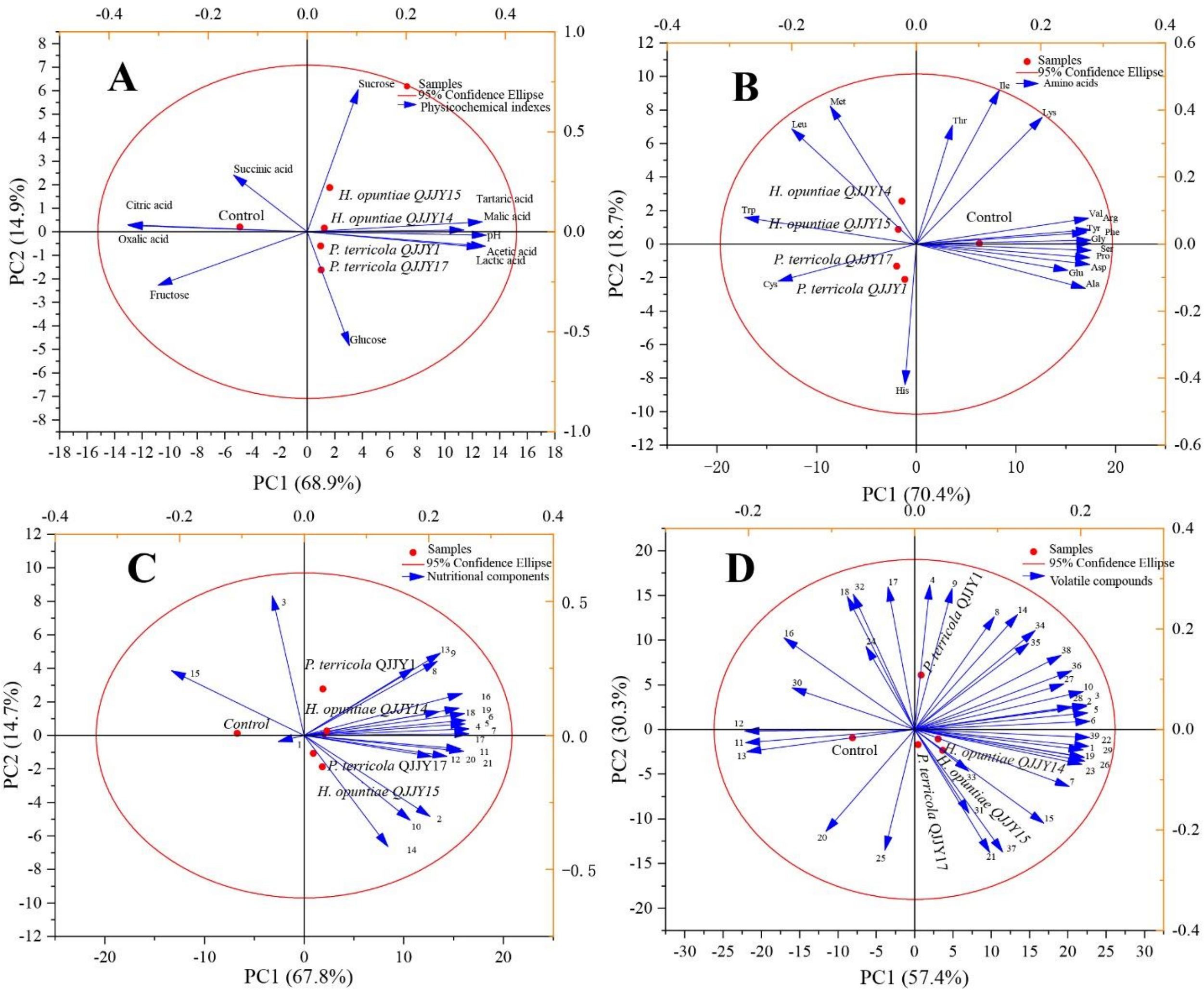

Supplementary Figure S1. Principal component analysis (PCA) of the physicochemical index (A), free amino acids (B), nutritional components (C), and volatile compounds (D). The numbers of the nutritional components in Figure C, such as 1, 2, and 3, correspond to those in Table 2. The numbers of the volatile compounds in Figure D, such as 1, 2, and 3, correspond to those in Table 3. Control: non-fermented whole Calamondin puree; fermented whole Calamondin puree: *P. terricola* QJJY1, *H. opuntiae* QJJY14, *H. opuntiae* QJJY15, or *P. terricola* QJJY17.

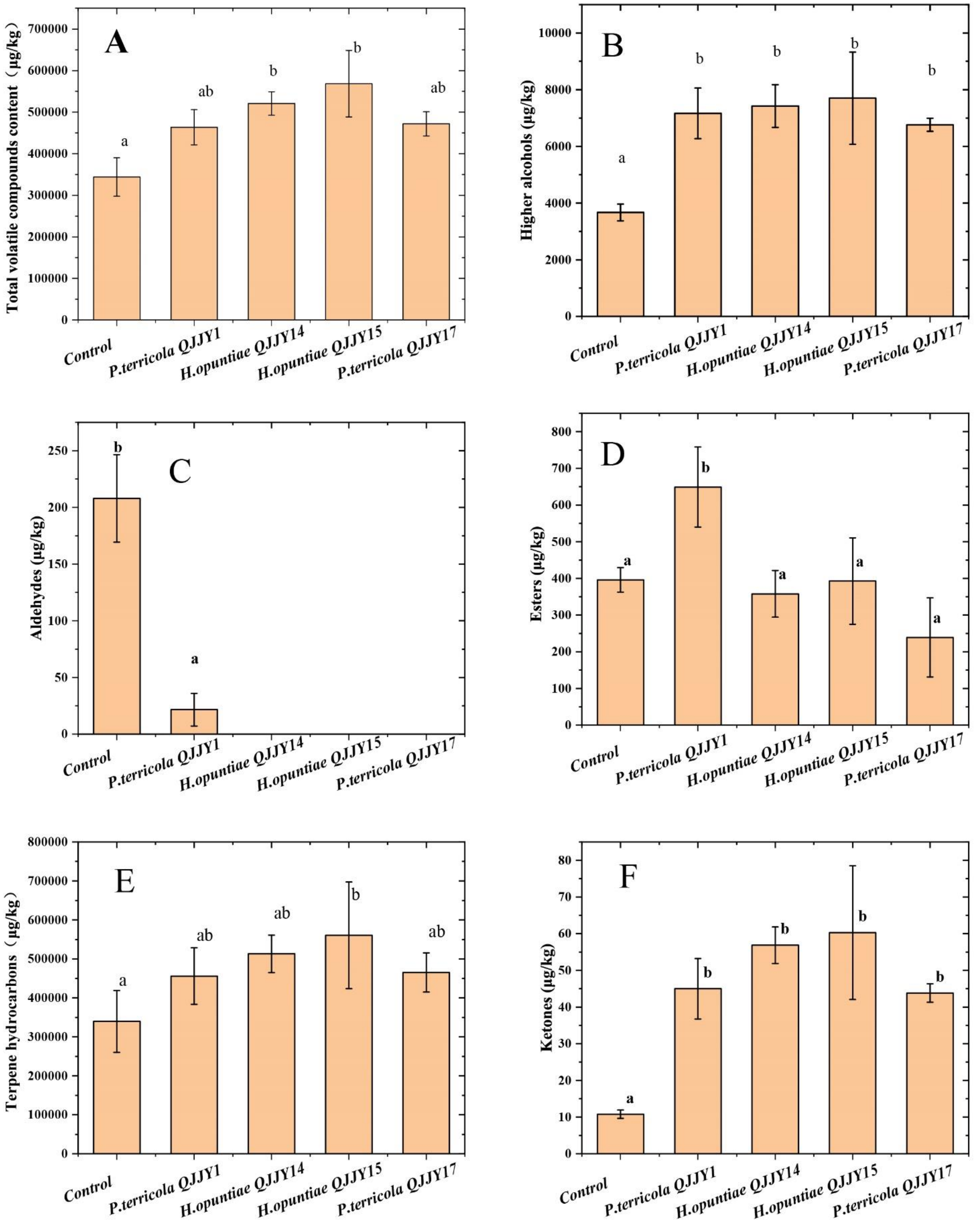

Supplementary Figure S2. Concentrations of volatile compounds in non-fermented (control) and fermented whole Calamondin puree (20 days) using *P. terricola QJJY1*, *H. opuntiae QJJY14*, *H. opuntiae QJJY15*, and *P. terricola QJJY17*. (A) Change in the total volatile compound content; (B) change in the higher alcohol content; (C) change in the aldehyde content; (D) change in the ester content; (E) change in the terpene hydrocarbon content; (F) change in the ketone content.

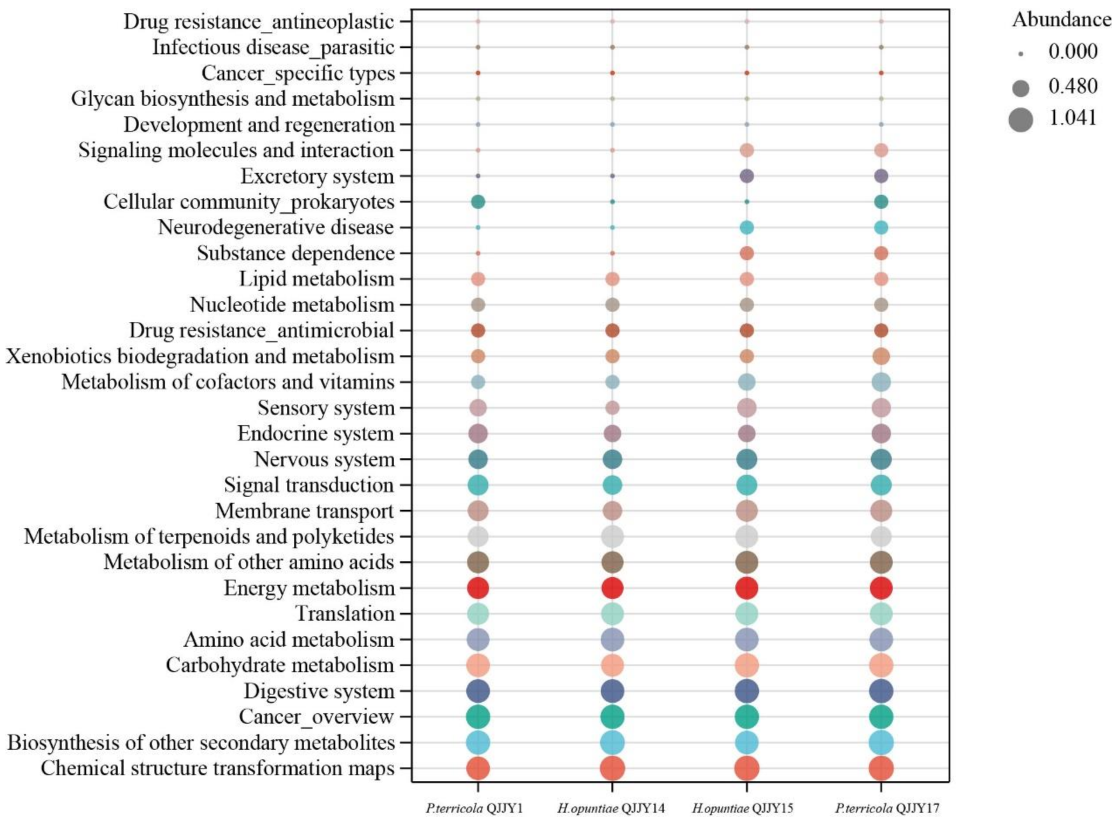

Supplementary Figure S3. Enrichment metabolic pathway bubble map of the characteristic differential metabolites in non-fermented (control) and fermented whole Calamondin puree (20 days) using *P. terricola QJJY1, H. opuntiae QJJY14, H. opuntiae QJJY15*, and *P. terricola QJJY17*. The number of different metabolites contained in each pathway was treated by log_{10}. The bubble size indicates the number of differential metabolites contained in the metabolic pathway; the larger the bubble size, the more abundant the metabolic pathways.
